# Supplementary material for: Functional Analysis of Spontaneous Cell Movement under Different Physiological Conditions
Source: PLoS One. 2008 Jul 9;3(7):e2648. doi: 10.1371/journal.pone.0002648 (PMC2444018; doi:10.1371/journal.pone.0002648)
Supplement: Figure S1 — Dependence of average cellular velocity and directness on developmental period. The definition of cellular velocity is described in the main text, and the temporal and ensemble average of the velocity in each developmental period is plotted. Directness of i-th cell is defined as cos θi, where θi is the angle between the direction of displacement of i-th cell and the direction of horizontal axis. Average directness of the cell population was calculated as N, where <>N is the ensemble average. Thus, in a randomly moving population of cells the value takes zero, while in a population of cells moving to a specific direction (horizontal axis) the value takes unity. Standard errors for average velocity and standard deviations for average directness are plotted together. (0.12 MB DOC) [file pone.0002648.s001.doc]

**Figure S1**


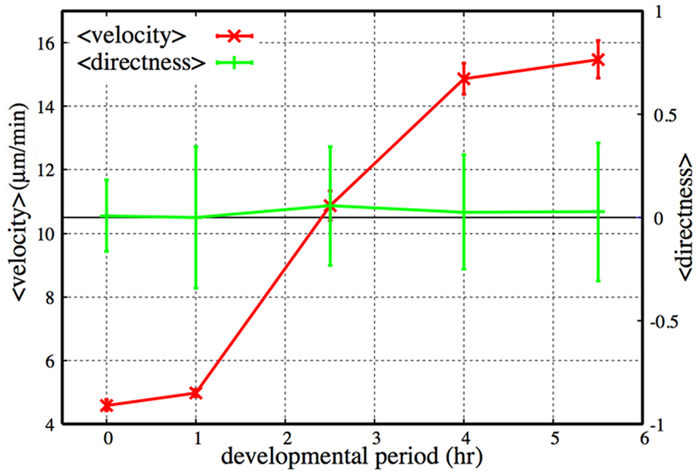


**Figure S1 legend**

Dependence of average cellular velocity and directness on developmental period. The definition of cellular velocity is described in the main text, and the temporal and ensemble average of the velocity in each developmental period is plotted. Directness of *i-th* cell is defined as , where is the angle between the direction of displacement of *i-th* cell and the direction of horizontal axis. Average directness of the cell population was calculated as , where is the ensemble average. Thus, in a randomly moving population of cells the value takes zero, while in a population of cells moving to a specific direction (horizontal axis) the value takes unity. Standard errors for average velocity and standard deviations for average directness are plotted together.
